# Comment on “Detecting Key Genes Regulated by miRNAs in Dysfunctional Crosstalk Pathway of Myasthenia Gravis”

**DOI:** 10.1155/2017/6950308

**Published:** 2017-02-08

**Authors:** Rozen Le Panse

**Affiliations:** Centre de Recherche en Myologie GH Pitié-Salpêtrière, 105 boulevard de l'Hôpital, 75013 Paris, France

I am concerned by an article “Detecting Key Genes Regulated by miRNAs in Dysfunctional Crosstalk Pathway of Myasthenia Gravis” by Cao et al., 2015 [1].

In this study, the authors used data from public databases to compare microarray data from the thymus and miRNome data from peripheral blood mononuclear cells (PBMCs). They analyzed the regulation of mRNAs from the thymus by miRNAs from peripheral blood mononuclear cells: two different “tissues.” Of course, they ended up with results but one can really wonder about the meaning of these results.

Moreover, in their manuscript, Cao et al. compiled thymic microarray data altogether while data are from different categories of patients (seropositive (for anti-AChR antibodies) and seronegative patients) (Le Panse et al., The Journal of Immunology, 2006) [2]. In AChR-myasthenia gravis patients, the thymus is involved in the disease but not in seronegative myasthenia gravis patients.
